# Pivotal Role of Intestinal Microbiota and Intraluminal Metabolites for the Maintenance of Gut–Bone Physiology

**DOI:** 10.3390/ijms24065161

**Published:** 2023-03-08

**Authors:** Niklas Grüner, Anna Lisa Ortlepp, Jochen Mattner

**Affiliations:** 1 Mikrobiologisches Institut—Klinische Mikrobiologie, Immunologie und Hygiene, Universitätsklinikum Erlangen and Friedrich-Alexander Universität (FAU) Erlangen-Nürnberg, 91054 Erlangen, Germany; 2 Medical Immunology Campus Erlangen, Friedrich-Alexander Universität (FAU) Erlangen-Nürnberg, 91054 Erlangen, Germany

**Keywords:** gut–bone axis, intestinal microbiota, intestinal dysbiosis, hormone and electrolyte metabolism, cell trafficking, inflammatory bowel disease, cytokines, arthritis, osteoporosis, spondyloarthropathy

## Abstract

Intestinal microbiota, and their mutual interactions with host tissues, are pivotal for the maintenance of organ physiology. Indeed, intraluminal signals influence adjacent and even distal tissues. Consequently, disruptions in the composition or functions of microbiota and subsequent altered host–microbiota interactions disturb the homeostasis of multiple organ systems, including the bone. Thus, gut microbiota can influence bone mass and physiology, as well as postnatal skeletal evolution. Alterations in nutrient or electrolyte absorption, metabolism, or immune functions, due to the translocation of microbial antigens or metabolites across intestinal barriers, affect bone tissues, as well. Intestinal microbiota can directly and indirectly alter bone density and bone remodeling. Intestinal dysbiosis and a subsequently disturbed gut–bone axis are characteristic for patients with inflammatory bowel disease (IBD) who suffer from various intestinal symptoms and multiple bone-related complications, such as arthritis or osteoporosis. Immune cells affecting the joints are presumably even primed in the gut. Furthermore, intestinal dysbiosis impairs hormone metabolism and electrolyte balance. On the other hand, less is known about the impact of bone metabolism on gut physiology. In this review, we summarized current knowledge of gut microbiota, metabolites and microbiota-primed immune cells in IBD and bone-related complications.

## 1. Introduction

The gastrointestinal tract hosts a broad range of highly diverse microorganisms, including viruses, parasites, fungi and bacteria. All of these microorganisms are referred to as intestinal microbiota. The number of intestinal microbiota might even outnumber the body’s own cells [1,2,3]. The interactions between commensal microbiota and the host’s own cells in the gut are pivotal for many physiological organ functions and the maintenance of the body’s immune homeostasis. Therefore, the composition of microbial species in the gut, and their combinational interactions with host tissues, are unique for each individual. Dietary, environmental and genetic factors of the host influence the composition and function of intestinal microbiota [4,5,6,7]. However, genes of different microbiota share overlapping and redundant functions [8,9], so that compositional changes of gut microbiota do not necessarily reflect metabolic and/or functional alterations. 

Microbiota play pivotal functions in the physiology of the host. They help to digest dietary products, contribute to the production of vitamins and produce—frequently in mutual concert with the host—various metabolites that play essential roles for the maintenance of organ physiology [10,11,12,13]. Moreover, intestinal microbiota are pivotal for the development of gut-associated lymphoid tissues and the induction of local and systemic immune responses [14,15]. Resident microbiota also regulate the development and differentiation of intestinal immune cell populations and shape the local cytokine milieu in the gut [16]. Microbiota of the gut are not only important for the development of gut-associated lymphoid tissue and the generation of certain immune cells, but also for the maintenance or restoration of non-inflammatory homeostasis in the intestine, attributable in part to microbial metabolites [16,17,18,19]. Additionally, the microenvironment of the tissue, as well as spatial and/or temporal interactions between the immune system of the host and the intestinal microbiota, shape the local immune response [20]. This latter could even exhibit situationally opposite effects. Various cytokines and immune cells also influence the composition and function of the intestinal microbiota. However, the mechanisms by which specific bacterial populations induce the development of individual cell subsets, or by which the cytokine milieu shapes the composition of the intestinal microbiota, remain poorly understood. 

Microbiota and their metabolites influence the local tissue environment of the gut. They also signal to distant tissues, such as the liver, the bone or the brain [12,21,22]. These physiological interactions can change significantly when the composition of the microbiota and/or the permeability of the intestinal epithelial barrier changes. Consequently, many disorders have been associated with intestinal dysbiosis. Indeed, leaky gut syndrome is associated with many inflammatory and immune-mediated diseases, including inflammatory bowel disease (IBD), type 1 diabetes (T1D), osteoporosis and arthritis [23,24,25,26,27]. Interestingly, although intestinal barrier dysfunction contributes to chronic, low-grade inflammation during aging, it has yet to be determined whether restoring barrier function is able to ameliorate clinical manifestations in gastrointestinal or systemic diseases [28,29]. Particularly, the role of the intestinal barrier in the gut–bone axis, under physiological and pathophysiological conditions, has become a focus of recent interest [24,28].

## 2. The Contribution of the Gut–Bone Axis to Bone Metabolism

**Figure 1:**
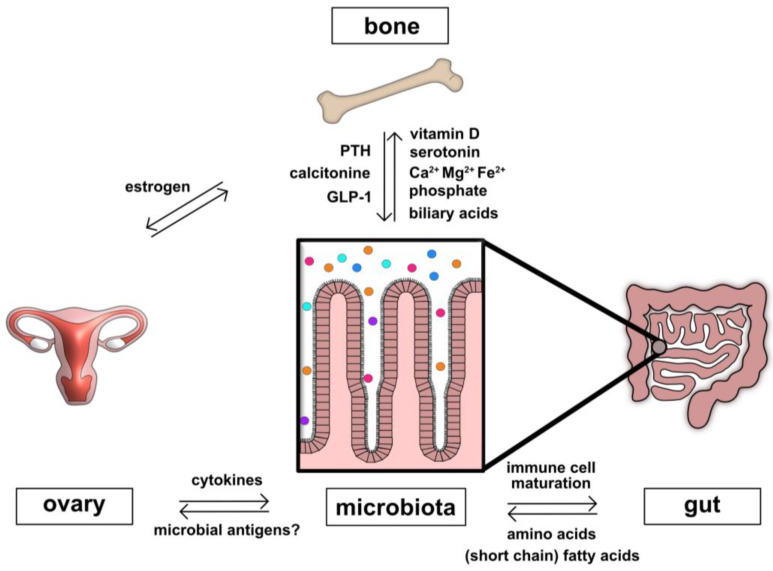
Influence of gut microbiota on physiological circuits regulating bone metabolism. Intestinal microbiota can interfere with several factors and signaling pathways regulating physiological bone homeostasis and metabolism. For example, calcitonin and PTH (hormones which exert opposite effects on mineralization) require intestinal microbiota to exert their anabolic and catabolic effects. Microbiota enzymatically activate estrogens, control cytokine production, and control immune cell differentiation, particularly Th17-Treg homeostasis. Intraluminal, microbiota-dependent amino acid and short chain fatty acid (SCFA) metabolism contributes (in part) to the regulation of these immune responses. Intestinal microbiota are crucial for the maintenance of the intestinal physiological barrier and electrolyte balance. Vitamin D is pivotal to establishing microbial diversity in the gut.

Interactions between the gut and bones are complex. Intestinal microbiota, for example, can interfere with various signaling circuits that regulate bone metabolism (Figure 1). We summarized, herein, some of the most important electrolytic, hormonal and metabolic factors that interfere with bone homeostasis, and described the pathways that microbiota influence. Electrolytes, like calcium and magnesium, or minerals like iron are pivotal for bone mineralization. Calcium intake influences skeletal calcium retention during growth and thus affects peak bone mass in early adulthood [30]. Moreover, magnesium intake is associated with higher hip and femoral neck bone mineral density [31]. Iron is essential for bone metabolism and has been positively related to bone mineral density [32]. On the other hand, disruptions of iron metabolism, leading to both iron overload and iron deficiency, are associated with low bone mineral density and fragility [33]. Iron deficiency anemia is another common systemic manifestation of IBD [34,35]. Microbiota also interfere with electrolyte and mineral metabolism. Thus, for example, specific intestinal microbiota can bind iron in the large intestine, limiting free radical formation. Certain probiotics affect calcium and phosphate resorption, as well, due to the regulation of the small intestinal barrier [36]. 

Vitamin D aids in the intestinal absorption of calcium. As it is synthesized in the skin or absorbed from the diet, vitamin D is converted, over several steps, into its active form. The active form, 1,25-dihydroxy vitamin D, subsequently interacts with the vitamin D receptor (VDR) to modulate the expression of target genes [37]. Thus, 1,25-dihydroxy vitamin D regulates bone and cartilage anabolism and the availability of calcium and iron for physiological tissue matrix mineralization [37,38]. Vitamin D supplementation increases the diversity of the gut microbiota, and vitamin D deficiency is associated with gut dysbiosis and inflammation [39]. 

Next to 1,25-dihydroxy vitamin D, there are two other hormones that play significant roles in electrolyte metabolism in the body, as well as the regulation of bone remodeling and density. These are parathyroid hormone (PTH) and calcitonin. Both are essential for the maintenance of mineral bone physiology, and both regulate calcium–phosphate metabolism. Low calcium levels trigger PTH production. Low and intermittent doses of PTH exhibit anabolic effects on bone volume and microarchitecture. In contrast, a continued hypersecretion of PTH, such as that which occurs in primary hyperparathyroidism, leads to bone resorption [40,41]. Acting as an opponent of PTH, calcitonin reduces calcium concentrations in the blood, for example, due to the inhibition of bone suppression or the suppression of calcium release from the bone [42]. Thus, calcitonin can retain bone density and reduce the risk of fractures. Both calcitonin and PTH require elements of the intestinal microbiota to exert catabolic and anabolic effects on bone metabolism. For example, many gut microbiota are able to digest carbohydrates to generate short chain fatty acids (SCFAs). These promote the differentiation of regulatory T cells (Tregs) from naive T lymphocytes [43,44,45]. Tregs mediate the bone anabolic activity of the probiotic *Lactobacillus rhamnosus* [46,47]. Thus, as Tregs interfere with PTH-induced bone metabolism [48], Treg differentiation is pivotal for the bone anabolic activity of PTH [49]. 

Another hormone that contributes to the growth and maturation of bones is estrogen. This steroid hormone regulates mammalian reproduction bone turnover in adult bones and promotes the proper closure of the epiphyseal growth plates during bone growth [50]. Decreased estrogen production in postmenopausal women is an underlying factor in the rapid bone resorption, resulting in osteoporosis, that many women experience [51,52]. Furthermore, estrogen deficiency can trigger the release of TNF-alpha, leading indirectly to bone loss and osteoclastogenesis [53]. The gut microbiota secretes an enzyme, beta-glucuronidase, which deconjugates estrogens into their active forms [54]. Thus, intestinal microbiota regulate the activity and functions of estrogen. 

The mutual crosstalk of the host and the intestinal microbiota also extends to other factors that are pivotal to bone homeostasis and skeletal microarchitecture [55]. Typically, microbiota promote catabolic effects on bone homeostasis [56] and regulate physiological bone turnover [57]. Furthermore, microbiota shape the local cytokine milieu and can affect bone metabolism via the release of cytokines. Particularly, the balance between Th17 and regulatory T lymphocytes (Tregs) is critical for bone metabolism [55]. Short chain fatty acids (SCFAs), released by microbiota upon carbohydrate digestion, are also important modulators of bone physiology [45,58,59,60]. We discussed the consequences of disruption in Th17 and Treg homeostasis and reduced SCFA production on bone physiology in more detail in Section 6.2 and Section 6.5. Serotonin and its precursor tryptophan are both primarily synthesized in the gut, and are therefore influenced by intestinal microbiota. They can regulate bone mass, and can also exhibit antagonistic functions, based on the body compartment in which they are acting [61].

Biliary acids mediate other secondary effects of microbiota. For example, following engagement of the farnesoid X receptor (FXR) and the G protein-coupled bile acid receptor 5 (TGR5), intestinal microbiota can change the amount and type of secondary bile acids [62]. TGR5 ligation by secondary bile acids can induce the production of glucagon-like peptide-1 (GLP-1), which in turn activates the proliferation of thyroid C cells and the secretion of calcitonin, thus, inhibiting bone resorption [63]. GLP-1 can also trigger the proliferation of osteoblasts and inhibit osteoclast activity [64]. Furthermore, monohydroxylated secondary lithocholic acid (LCA) can serve as a direct ligand for the vitamin D receptor [65]. Thus, intestinal microbiota are crucial for directly and indirectly regulating the gut–bone axis. Consequently, intestinal dysbiosis can disrupt bone physiology. One of the pathological conditions that can simultaneously affect the gut and the bone is inflammatory bowel disease (IBD). Therefore, we discussed osteoporosis and arthritis as separate disorders—and as two common extraintestinal disease manifestations of IBD patients.

## 3. Compositional Changes of the Intestinal Microbiota as Signature of IBD

Epithelial surfaces, such as the gut, form a large interface between the body and the external environment. An immune-mediated inflammation of the gastrointestinal (GI) epithelium characterizes inflammatory bowel diseases (IBD), such as Crohn´s disease (CD) or ulcerative colitis (UC) [66,67]. Clinical relapses, as well as severe gastrointestinal mucosal and transmural lesions in a non-sterile environment, characterize both disorders [68,69]. Although the exact etiologies underlying IBD remain elusive, these disorders presumably result from a complex interplay of microbial, genetic, geographic and habitual factors, which consequently disrupts interactions of intestinal microbiota, the intestinal epithelium and the immune system [70,71,72]. Various cellular, cytokine and molecular pathways perpetuate immune dysfunction in IBD [71,73,74]. Biologicals targeting these pathways, at best, induce remission in just half of the patients [75,76,77]. 

Intestinal microbiota are critical components in the pathogenesis of IBD, as they influence nutrient metabolism and host immune responses. IBD patients exhibit an altered composition of gut microbiota compared to healthy individuals [78,79]. Increasing evidence implies that the microbiota initiate and maintain intestinal inflammation [80]. Common pathogenic mechanisms include an increased exposure of bacterial antigens to intestinal immune cells of the host and/or alterations in the immune response of the host to commensal bacteria [81,82,83,84,85]. Whether these alterations in the composition of the intestinal microbiota are the consequence, or the cause, of intestinal inflammation has become a subject of intensive investigation. Fecal microbiota transfers (FMTs) from healthy donors, replacing dysbiotic microbiota in IBD recipients, could be a novel treatment for IBD [17,18,19,86,87,88,89]. 

## 4. The Bone as Frequent Target of Extraintestinal IBD Manifestations

**Figure 2:**
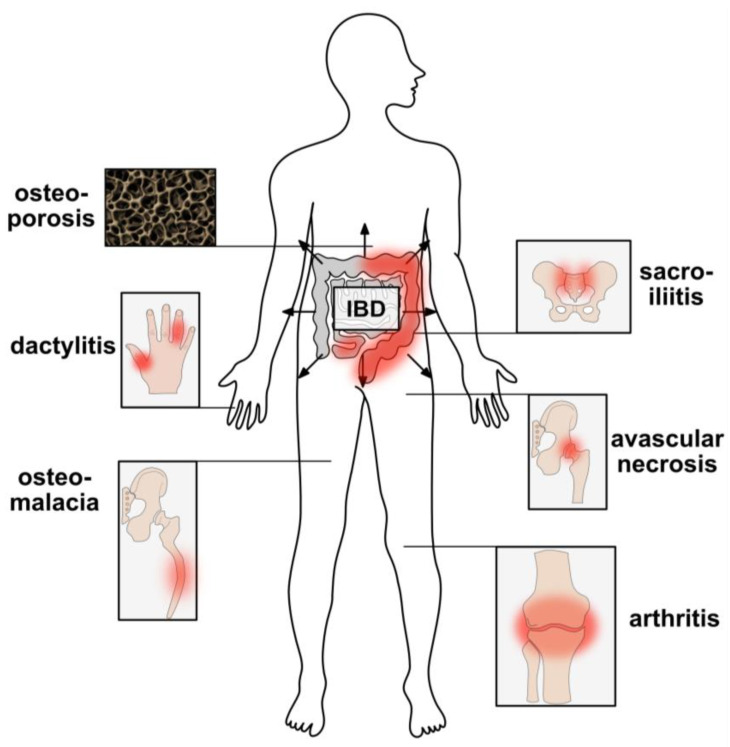
Extraintestinal disease manifestations of IBD affecting bones and joints. Involvements of the joints experienced concomitantly with IBD include arthritis, sacroiliitis and dactylitis, as well as disease manifestations affecting the complete bone, such as osteoporosis, osteomalacia and avascular necrosis.

Diarrhea, rectal bleeding, abdominal pain, fatigue and weight loss accompany both UC and CD [72,90,91]. Typically, periods of active disease follow periods of remission. Depending on the severity of inflammation and the affected parts of the gut, symptoms vary and may range from mild to severe. Furthermore, IBD patients frequently experience extraintestinal manifestations [92,93] and complain about musculoskeletal, ocular and/or cutaneous symptoms in addition to gastrointestinal symptoms. Commonly, these extraintestinal symptoms (which occur in 5% to 50% of all IBD patients) result in significant morbidity, even more than intestinal disease itself [94]. Impacts on the musculoskeletal system are among the most common extraintestinal manifestations; osteoporosis and arthritis are two common clinical problems accompanying IBD [94,95] (Figure 2). 

Enteropathic arthritis, associated with IBD, is distinct from rheumatoid arthritis; it belongs to an extended disease group of inflammatory arthritides, namely the spondyloarthropathies (SpA). These share common clinical features, including axial spondylitis, sacroiliitis, peripheral inflammatory arthritis, enthesitis and dactylitis [95,96,97,98,99,100]. Additional extraintestinal manifestations of IBD affecting bone and joints include ankylosing spondylitis, avascular hip necrosis and osteomalacia [94] (Figure 2). Although most extraintestinal manifestations, including pauci-articular arthritis, are directly associated with ongoing intestinal disease flares, others, like ankylosing spondylitis, occur independently of intestinal disease activity [94].

### 4.1. Osteoporosis/Osteopenia 

Bone is a living tissue that undergoes constant remodeling by bone-forming cells (osteoblasts) and bone-resorbing cells (osteoclasts). Imbalances in osteoblastic bone formation and osteoclastic bone resorption lead to osteopenia or osteoporosis [101,102,103,104]. Both osteopenia and osteoporosis are quantitative, rather than qualitative, metabolic disorders of bone mineralization [105]. 

A decrease in bone mineral density (BMD) characterizes both disorders. Osteopenia shows BMD scores below normal reference values, but not low enough to meet the diagnostic criteria for osteoporosis [104]. Thus, the extent of bone microarchitecture disruption makes it possible to distinguish one disease from the other [104,105]. The more pronounced the bone loss, the more fragile the bone becomes, resulting in increased risk of fracture and disability [103].

Both osteoporosis and osteopenia are multifactorial diseases [103]. Endocrine mechanisms, such as estrogen or vitamin D deficiency, as well as secondary hyperparathyroidism, underlie their pathogeneses, similarly to dysregulation in interactions between bone and the immune system, dysbiosis of the gut microbiota and cellular senescence [103,104,105]. Indeed, some of the same pathogenic mechanisms underlie both IBD and osteopenia/osteoporosis. 

### 4.2. Influence of Intestinal Microbiota on Postmenopausal Osteoporosis

Osteopenia and osteoporosis are common metabolic bone diseases in postmenopausal women. Postmenopausal cessation of ovarian function—and the consequent estrogen deficiency—is the primary cause of both metabolic bone diseases (Figure 1). Declining estrogen levels result in the stimulation of bone resorption and—to a lesser extent—bone formation, leading to a period of rapid bone loss [106].

A growing body of evidence suggests that the gut microbiota are involved in the regulation of bone metabolism [63,107,108]. In comparisons of healthy postmenopausal women with women suffering from osteopenia and osteoporosis, the composition of microbiota significantly differed. While the intestinal microbiota of healthy controls contained more *Clostridia* and *Methanobacteriaceae*, more *Bacteroides* were recovered from the feces of osteopenic and osteoporotic women [109]. Another study confirmed alterations in the composition of intestinal microbiota in postmenopausal women. The authors observed an accumulation of *Fusicatenibacter*, *Lachnoclostridium*, and *Megamonas* spp. in women with osteoporosis, along with an increase of TNF-alpha serum levels and a decrease of serum IL-10 concentrations. Although they observed additional alterations in the composition of vaginal microbiota, they concluded that compositional changes in the intestinal microbiota were more closely correlated to osteoporosis [110]. 

In mice, ovariectomy (ovx) or treatment with gonadotropin-releasing hormone (GnRH) agonists modeled the effects of estrogen depletion [111,112]. Microbiota promoted osteoporosis in sexual-hormone-deficient mice. However, dietary supplementation of specific probiotics, such as *Lactobacillus rhamnosus*, protected mice from osteoporosis by strengthening the barrier integrity of the gut and, consequently, alleviating inflammation [113]. Accordingly, fecal microbiota transplants (FMTs) protected recipients from osteoporosis as well. A reduced production of osteoclastic cytokines, such as TNF-alpha or IL-1beta, and an increase in SCFAs accompanied that protection [114]. Moreover, FMTs strengthened the intestinal barrier, as demonstrated by increased expression of tight junction proteins. 

### 4.3. Osteoporosis in IBD

Several gastrointestinal disorders have been associated with osteoporosis and osteopenia, including IBD, celiac disease and chronic liver disease [115,116,117,118]. Indeed, osteoporosis and arthritis are leading causes of morbidity in IBD patients [94]. Bone loss is an early systemic process and can occur even before clinical disease manifests. Thus, extraintestinal symptoms affecting the bone need to be considered in IBD therapy.

IBD has been associated with decreased bone mass and alterations in bone geometry from the time of diagnosis, frequently before the initiation of anti-inflammatory therapy. Bone disease is attributed to vitamin D deficiency, steroid use and/or systemic inflammation [119]. IBD patients are at higher risk for developing osteoporosis and osteopenia than the general population, with a 40% higher relative risk of fracture in IBD patients [120,121,122]. The prevalence of osteopenia and osteoporosis in IBD patients varies significantly depending on the study populations, location and design, ranging from 22% to 77% and from 17% to 41%, respectively [122,123,124]. 

The etiology of osteoporosis in IBD is multifactorial, with risk factors including age, (long-time) corticosteroid use, (protein-calorie) malnutrition, vitamin D and calcium malabsorption and deficiency, immobilization and the underlying inflammatory state [120,121,122,125,126]. Inactivity, hypogonadism and stunted growth in children, as well as decreased skeletal muscle mass, also most likely play a role in the pathogenesis. Deficits in bone mass can persist despite the absence of symptoms of active IBD.

**Figure 3:**
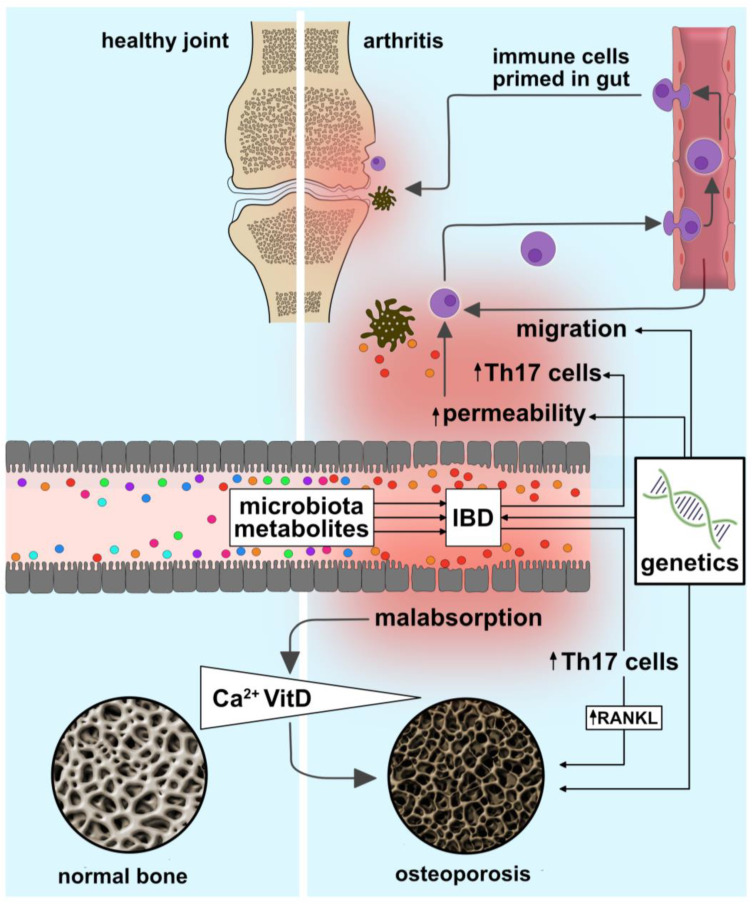
Pathogenic mechanisms underlying bone disease in IBD. IBD is a multifactorial disease driven by environmental factors and complex genetic traits. Gut microbial dysbiosis, intestinal barrier disruption, dysregulated intraluminal metabolism and aberrant immune responses characteristically accompany IBD. For example, dysbiosis-driven immune cell priming mitigates the expansion, the egress and the migration of Th17 cells from the gut into the joints, causing arthritis. Enhanced angiogenesis further promotes inflammatory cell and cytokine transport. Both the inflammation-associated disruption of the intestinal barrier and dysbiotic microbiota can lead to a malabsorption of calcium and disruptions of vitamin D metabolism, underlying the development of osteoporosis.

The effects of IBD on the skeleton are complex. Preliminary studies suggested that the dysbiotic intestinal microbial flora present in IBD could also affect bone at a distance. Several mechanisms underlying dysregulated gut–bone interactions are possible. For example, T cells activated by the gut microbiota may serve as inflammatory shuttles between the intestine and the bone (Figure 3). Microbe-associated molecular patterns leaked into the circulation in IBD could activate immune responses in the bone marrow by immune cells, osteocytes, osteoblasts and osteoclasts, leading to decreased bone formation and increased resorption. Finally, intestinal microbial metabolites, such as hydrogen sulfide (H_2_S), may also impair bone cell functions. Uncovering these mechanisms will enable the design of microbial cocktails to help restore bone mass in IBD patients [127]. 

Accordingly, in almost 15% of IBD patients that were analyzed in a prospective, single center study, markers for bone resorption, such as the C-terminal telopeptide of type 1 collagen (CTX), were increased. Bone mineral alterations were common in IBD patients and Vitamin D supplementation was found to be crucial, especially when taking corticosteroids, azathioprine and/or infliximab [119]. Interestingly, the severity (but not the activity) of disease was associated with osteopenia in IBD patients [128,129,130]. Furthermore, patients suffering from IBD in their younger years had a lower bone density compared to healthy controls [131,132]. Moreover, IBD patients are at increased risk for fractures. Particularly, young IBD patients with high inflammatory burdens or corticosteroid exposure may suffer from bone mineral loss and exhibit a higher risk of fractures in later years. An earlier, more aggressive therapy to reduce inflammation and/or the use of steroid-sparing drugs could reduce the risk. Furthermore, IBD patients should have their bone density checked frequently [133].

### 4.4. Arthritis and Spondyloarthropathies in IBD

Significant numbers of patients with spondyloarthropathies (SpA) suffer from associated clinical IBD. Nearly half of them show subclinical gut inflammation; however, the connection between the gut and the musculoskeletal system has remained an exasperating problem [96]. Musculoskeletal symptoms include the most common extraintestinal manifestations of IBD. Peripheral arthritis is the most common extraintestinal manifestation in CD and UC patients [134]. An observational two-year cross-sectional study reported that 30% of IBD patients with musculoskeletal complaints for more than three months had SpA. SpA occurred more frequently in men and patients with surgery for IBD [135]. They accounted for about 40% of all extraintestinal manifestations and manifest mainly as axial or peripheral spondyloarthritis. Spondylitis ankylosans was found in 5–10% of IBD patients. About 25% of IBD patients suffered from sacroiliitis [94] (Figure 2).

Another study confirmed arthritis as the most common extraintestinal complication of CD and UC. Indeed, more than 21% of CD patients and 12.5% of UC patients suffered from arthritis, suggesting that arthritis was even more frequent in CD than in UC [136]. Prevalence of extraintestinal manifestations in the locomotor system decreased with age, with a prevalence of almost 25% in 20–30 year old patients, down to 2% in 50–60 year old patients. Peripheral arthritis occurred in 5–14% of UC patients and in 10–20% of CD patients [93]. 

## 5. Animal Models of Inflammatory Bone Loss 

The close relationship between inflammation and bone metabolism has been appreciated and recognized for a long time [66,81,137]. Toll-like receptors (TLRs) play pivotal roles in inflammation and provide important links between the immune and skeletal systems. In a study, TLR9^−/−^ mice exhibited low bone mass and low-grade systemic chronic inflammation, which as characterized by the expansion of CD4^+^ T cells and increased levels of inflammatory cytokines, including TNF-alpha, RANKL (in detail described below) and IL-1beta. The increased levels of these cytokines significantly promoted osteoclastogenesis and induced bone loss. Importantly, TLR9 deletion altered the gut microbiota, and this dysbiosis was an underlying factor in the systemic inflammation and bone loss observed in TLR9^−/−^ mice [138]. 

Short chain fatty acids (SCFAs ) are microbiota-derived metabolites that can potentially influence bone homeostasis. They mainly consist of acetate, butyrate and propionate. Indeed, these products of dietary fibers enhance alkaline phosphatase activity, a marker of osteoblast differentiation, in cell lines in vitro. Thus, SCFAs might contribute to the maintenance of a positive balance of bone turnover [58]. SCFAs also regulate osteoclast metabolism and bone mass in vivo. Treatment of mice with SCFAs or feeding with a high-fiber diet significantly increased bone mass and prevented postmenopausal and inflammation-induced bone loss. The protective effects of SCFAs on bone mass were primarily associated with inhibition of osteoclast differentiation and bone resorption, while bone formation was less affected [59]. 

The Winnie mouse, which carries a mutation in the *Muc2* gene and serves as a model of spontaneous chronic colitis, also exhibited colitis-associated bone loss under study. Compared to B6 controls, these defects included a deterioration in trabecular and cortical bone microarchitecture, increased bone resorption, and decreased bone formation and bone strength. Moreover, the number of osteoblasts decreased, while the number of osteoclasts increased. The onset and progression of intestinal inflammation were associated with increased gut-derived serotonin levels. Thus, the skeletal phenotype of Winnie mice closely resembled the clinical manifestations of IBD-associated osteoporosis/osteopenia [139]. 

## 6. Dysregulated Gut–Bone Interactions Potentially Underlying Skeletal Disease Manifestations in IBD

As outlined above, the bone is a frequent target of extraintestinal IBD manifestations. Multiple mechanisms might underlie pathological alterations in the gut–bone axis and the subsequent induction of bone disease. These include intestinal dysbiosis, disrupted cytokine and chemokine responses, a deterioration of intestinal barrier integrity, aberrant vessel formation and metabolic alterations. Below, we discussed several of these in greater detail. 

### 6.1. Intestinal Dysbiosis

By comparing microbiota in healthy individuals with IBD patients, patients with ankylosing spondylitis (AS) or patients suffering from both, it was noted that the composition of microbiota from AS and IBD patients differed from healthy individuals [140]. Moreover, alpha- and beta-diversity were altered. *Streptococcus* and *Haemophilus* accumulated in both IBD and AS patients. However, the authors noted that microbiota might not have been underlying the pathogenesis of either disease, because microbiome composition varied along with different geographic regions independently of the disease status. This indicated that other factors could introduce bias into studies of the associations between microbiome composition and disease activity. Another study also detected alterations in the composition of microbiota. Therein, *Clostridiaceae* increased among the intestinal microbiota of patients with IBD and rheumatoid arthritis (RA) [141]. In another study, the authors speculated that molecular similarities between antigenic epitopes of gut microbiota and host tissues could possibly have induced cross-reactivity of T cells. Moreover, the increased epithelial barrier permeability could have been an underlying factor in the extraintestinal manifestations of IBD and systemic inflammatory reactions. Dysbiosis could therefore have activated intestinal immune cells and induced their migration to other organs outside the gut [93]. However, the specific microbiota underlying these pathogenic effects were difficult to define. Although another study confirmed alterations in the composition of intestinal microbiota, they observed an accumulation of other bacterial species. The numbers of *Actinomyces*, *Eggerthella*, certain *Clostridia*, *Faecalicoccus* and *Streptococcus* significantly increased in immune-mediated inflammatory diseases. CD patients exhibited the greatest variability in the composition of microbiota [142]. Consequently, there were microbiota that accumulated in both IBD patients and patients with chronic rheumatic diseases, such as *Bifidobacterium*, *Staphylococcus*, *Enterococcus*, *Lactobacillus*, *Pseudomonas*, *Klebsiella* and *Proteus*. In contrast, *Faecalibacterium* and *Roseburia* decreased in both disorders. However, some bacteria, such as *Eubacterium*, *Clostridium*, *Ruminococcus* and *Coprococcus*, specifically increased in chronic rheumatic disease, but decrease in IBD [142]. 

### 6.2. Cytokines

Antigens derived from gut microbiota are key targets for intestinal effector T cell activation and differentiation [143,144]. Particularly, the balance between T helper 17 (Th17) and regulatory T lymphocytes (Tregs) is critical for bone metabolism [55]. Indeed, disruptions of this Th17-Treg balance promote both intestinal and joint inflammation [145], and the Th17 pathway has been linked to both intestinal and joint disease [145]. Dysregulated microbiota drive Th17 cell expansion and immune cell migration to the joints [96,146,147,148] (Figure 3). Segmented filamentous bacteria (SFB), for example, enable parathyroid hormone (PTH) to expand intestinal TNF-alpha- and IL-17-producing T cells and promote their egress from the gut. Following migration into the bone marrow, Th17 cells induce the production of the receptor activator of nuclear factor kappa-Β ligand RANKL by osteoblasts and osteocytes, causing loss of bone mass [149]. RANKL controls bone regeneration and remodeling. Following stimulation with TNF-alpha or IL-17, a variety of different cell populations, including T- and B-lymphocytes and osteoblasts, release this primary driver of osteoclastogenesis [53,150,151,152]. Macrophage colony-stimulating factor (M-CSF) is the cytokine that initiates early osteoclast differentiation [72]. Vitamin D receptor negatively regulates bacterial-stimulated NF-kappa B activity in the intestine, demonstrating a reciprocal interaction between calcium homeostasis and microbiota [153]. 

Thus, alterations of the microbiota might change the antigen pattern and, consequently, the cytokine response. Inflammatory mediators such as IL-6, TNF-alpha, IFN-gamma and vascular endothelial growth factor (VEGF) are elevated in the serum of IBD patients. These systemic inflammatory responses can activate the production of cytokines in non-intestinal organs [92].

### 6.3. Intestinal Barrier Integrity and Immune Cell Trafficking

An impaired intestinal barrier precedes clinical diagnosis of IBD by years [154,155]. Intestinal microbiota and diurnal variations in diet microbiota interactions might be regulators of the intestinal barrier [156]. Under study, IBD patients suffering from concomitant spondyloarthritis exhibited enhanced epithelial permeability in the ileum. Expression of the tight junction proteins occludin and claudin-1/-4 was lowered, but there was an increase in bacterial infiltrates in the gut wall. Furthermore, these patients had enhanced levels of serum proteins such as I-FABP, LPS and sCD14 in their sera, indicating interactions with intestinal microbiota [157]. 

Along with the findings regarding altered intestinal permeability, a number of unique lymphocyte populations expand within the gut and the skin of patients with SpA, including gamma/delta T cells, mucosa-associated invariant T (MAIT) cells, innate lymphoid cells (ILCs) and T resident memory (TRM) cells. These cells respond to microbial cues at their barrier surface, causing cellular activation and generation of interleukin (IL)-17, which is hypothesized to be the mechanism by which the cells contribute to SpA pathogenesis [158]. Using photolabeling gut–joint trafficking of intraepithelial lymphocytes (IELs) to joint enthesis, the pathogenic site of SpA has been visualized [159]. Moreover, different adhesion molecules that mediate immune cells, homing in on the gut and joints, have been uncovered. The binding of intestinal lymphocytes to inflamed synovium depends on vascular adhesion protein-1 (VAP-1)—but not alpha4 beta7–MAdCAM-1—interactions [160]. The adherence of macrophages is mainly P-selectin dependent [161]. Gut-derived mucosal immune cells of IBD patients can bind to inflamed synovial venules, as well. Lymphocytes, for example, use VAP-1, CD44, CD18 integrins, intracellular adhesion molecule-1 (ICAM-1), L-selectin and peripheral lymph node addressins (PNAd), as well as interactions between alpha4 integrins and vascular cell adhesion molecule-1 (VCAM-1), to bind to synovial tissues. Mucosal macrophages, on the other hand, utilize P-selectin, and its ligand, P-selectin glycoprotein ligand-1 (PSGL-1), as well as E-selectin and VAP-1, for interactions with the synovium [162].

### 6.4. Angiogenesis

Gut microbiota can also affect the vascular system. Patients with acute coronary syndrome, for example, exhibited distinct serum metabolome and gut microbial signatures, as compared to control individuals [163]. Even in healthy individuals, lower gut microbial diversity was associated with higher white blood cell counts and C-reactive protein levels [164]. Moreover, patients with Behcet´s disease, a systemic autoimmune inflammation of the blood vessels, exhibited a different composition of intestinal microbiota, compared with healthy adults [165]. Gut dysbiosis, with elevated production of IL-6, IL-1beta, TNF-alpha and VEGF exacerbated pathological angiogenesis [166]. 

Similar microbiota-dependent effects likely play a role in the pathogenesis of IBD, as the intestinal vascular endothelium builds a second pivotal barrier next to the intestinal epithelium in the gut [74,167]. Gut microbiota can facilitate vascular dysfunction and hypertension, at least in part, due to MCP-1-/IL-17-driven vascular immune cell infiltration and inflammation [168]. Furthermore, inflammation activates angiogenesis in IBD [74,169,170,171]; dysfunction of the vascular barrier represents another important pathomechanism of IBD [74]. Microbiota contribute to the maintenance of vascular tone via actin polymerization [172]. Moreover, microbiota influence the enteric nervous system [173], which regulates intestinal permeability and blood vessels [174,175,176,177,178]. 

Similarly to IBD, arthritis is an angiogenesis-dependent disease. Angiogenesis is an early event in the inflammatory joint. It allows activated immune cells to enter the synovium [179]. Moreover, vascular turnover increases in the arthritic joint [55]. Increased vascular proliferation and/or blood flow could contribute to gut–joint trafficking of immune cells. Thus, the inflamed intestinal vasculature could provide novel therapeutic targets for the treatment of intestinal and extraintestinal IBD symptoms.

### 6.5. Metabolic Alterations

Similarly to what has been observed in osteoporosis, SCFAs protect against collagen-induced arthritis. In an experimental model of rheumatoid arthritis (RA), free fatty acid receptor 2 (FFA2) on CD19+ B cells underlay the protective effects [60]. TNF-alpha-overexpressing mice spontaneously developed IBD and RA, confirming the pivotal role of microbiota and microbiota-derived products in the pathogenesis of both diseases. FMTs of TNF-alpha-overexpressing donors into germ-free mice also induced IBD and RA in the recipients. The induction of autoreactive T cells due to dysbiotic gut microbiota could therefore represent a disease-mediating mechanism [180]. 

IBD and RA are also characterized by disrupted amino acid metabolism [96,181,182]. We briefly discussed this disrupted interaction with respect to the semi-essential amino acid L-arginine. For example, IBD patients exhibited altered availability of L-arginine in different tissue compartments [183,184,185]. Along with the reduced intraluminal L-arginine levels, a dietary L-arginine supplementation protected against DSS-, oxazolone- and *Citrobacter*-induced colitis in preclinical models [186,187,188,189]. Accordingly, an upregulation of the expression of the argininosuccinate lyase (ASL), the only enzyme able to produce L-arginine, was correlated with improved epithelial integrity and alleviation of colitis [190,191]. In contrast to the inflamed gut tissues, L-arginine concentrations increased in the plasma of IBD and RA patients, and could potentially mediate systemic adverse effects or disease [183,192]. Indeed, L-arginine might promote experimental arthritis, as signs of inflammation inversely correlated with the availability of L-arginine [192]. Accordingly, L-arginine deficiency, in multinucleated giant cells, improved symptoms of arthritis, as indicated by reduced swelling and weight loss [193]. In another study, dietary L-arginine application ameliorated experimental arthritis [194]. Consequently, the availability of L-arginine in distinct cellular and tissue compartments is critical for the influence of immune responses in both diseases. How this links IBD and RA together will be the subject of future investigations. 

Another example of altered metabolic gut–bone interactions is the gut microbiota-related metabolite trimethylamine N-oxide (TMAO). TMAO is a biologically active molecule, and is putatively involved in the promotion of different chronic diseases in humans [195]. In contrast, a reduction of plasma TMAO levels was associated with a greater loss of bone mineral density (BMD) in patients with type 2 diabetes [196]. Thus, as type 2 diabetes is related to obesity and altered bone health, by applying diets aiming for weight loss, thereapies must also consider adequately balancing TMAO and its precursors, choline and l-carnithine, as TMAO could prevent BMD reduction.

## 7. Conclusions

Intestinal microbiota and metabolites are pivotal for the maintenance of gut–bone physiology. They have been proven able to influence the absorption of calcium, magnesium and iron, to affect hormone metabolism and to directly and indirectly influence barrier integrity and immune responses. Specific probiotics, such as *Lactobacilli* or *Bifidobacteria*, regulate bone health [55,197]. Dysbiosis, on the other hand, leads to bone pathology in several ways—for example, reduced electrolyte absorption, altered metabolism, the expansion, egress and joint trafficking of Th17 cells, pathological angiogenesis and/or a disrupted barrier permeability. Clinical remission, endoscopic healing, absence of disability and a restoration of quality of life are the most important long-term achievable treatment targets [198]. These are most frequently achieved by potent anti-inflammatory drugs, e.g., antibodies like Adalimumab or Infliximab [92], or locally active or systemic therapies, with significant side effects [199]. Thus, alternative therapeutic approaches targeting intestinal and extraintestinal symptoms are urgently required. It would be intriguing to treat IBD by changing gut microbiota and intraluminal metabolites using simple dietary supplements. For instance, probiotics might induce remission in UC patients for a limited time [200]. Another method to preserve or reconstitute the gut microbiome is fecal microbiota transplantation (FMT). This has been well-established, and has demonstrated a very high degree of efficacy in patients suffering from refractory *Clostridioides difficile* infection (CDI). It also has a very good safety profile [201]. FMT has been studied in mice with experimental UC, where it improved Th1/Th2 and Th17/Treg imbalances through the regulation of intestinal microbiota [202]—which also affected musculoskeletal manifestations of IBD. In UC patients, FMT has shown promise, significantly improving rates of clinical remission in a recent meta-analysis [203]. Thus, it will be interesting to explore whether FMTs could also cure extraintestinal manifestations. Further research will hopefully clarify the mechanisms of inflammation-induced bone loss in IBD and guide effective treatment modalities [204]. In addition to FMTs, these could include the reconstitution of distinct microbial metabolites, such as SCFAs.

## Data Availability

All data are available in the presented manuscript.

## Abbreviations

AS	ankylosing spondylitis
ASL	argininosuccinate lyase
BMD	bone mineral density
CD	Crohn’s disease
CTX	C-terminal telopeptide of type 1 collagen
FMT	fecal microbiota transplant
FXR	farnesoid X receptor
GI	gastrointestinal tract
GLP-1	glucagon-like peptide-1
GnRH	gonadotropin-releasing hormone
H_2_S	hydrogen sulfide
IBD	inflammatory bowel disease
ICAM-1	intracellular adhesion molecule-1
IELs	intraepithelial lymphocytes
IFNgamma	interferon gamma
IgA	Immunoglobulin A
IL-10	interleukin 10
IL-6	interleukin 6
IL1-beta	interleukin 1 beta
ILCs	innate lymphoid cells
LCA	monohydroxylated secondary lithocholic acid
LPS	lipopolysaccharide
M-CSF	macrophage colony stimuation factor
MAIT	mucosa-associated invariant T cell
MCP-1	monocyte chemoattractant protein 1
Ovx	ovarectomy
PNAd	peripheral lymph node addressins
PSGL-1	P-selectin glycoprotein ligand-1
PTH	parathyreoid hormone
RA	rheumatoid arthritis
RANKL	Receptor activator of nuclear factor kappa-Β ligand
SCFA	short chain fatty acid
SFB	Segmented filamentous bacteria
SpA	spondyloarthropathies
T1D	Typ 1 Diabetes
TGF-beta	transforming growth factor beta
TGR5	G protein-coupled bile acid receptor 5
Th17	T helper 17 cell
TLR	Toll-like receptor
TMAO	trimethylamine N-oxide
TNF-alpha	tumor necrosis factor alpha
Tregs	regulatory T cells
TRM	T resident memory cell
UC	ulcerative colitis
VAP-1	vascular adhesion protein-1
VDR	vitamin D receptor
VEGF	vascular endothelial growth factor

## References

[1] Savage D.C. (1977). Microbial ecology of the gastrointestinal tract. Annu. Rev. Microbiol..

[2] Sender R., Fuchs S., Milo R. (2016). Are We Really Vastly Outnumbered? Revisiting the Ratio of Bacterial to Host Cells in Humans. Cell.

[3] Sender R., Fuchs S., Milo R. (2016). Revised Estimates for the Number of Human and Bacteria Cells in the Body. PLoS Biol..

[4] Aschard H., Laville V., Tchetgen E.T., Knights D., Imhann F., Seksik P., Zaitlen N., Silverberg M.S., Cosnes J., Weersma R.K. (2019). Genetic effects on the commensal microbiota in inflammatory bowel disease patients. PLoS Genet..

[5] Koutoukidis D.A., Jebb S.A., Zimmerman M., Otunla A., Henry J.A., Ferrey A., Schofield E., Kinton J., Aveyard P., Marchesi J.R. (2022). The association of weight loss with changes in the gut microbiota diversity, composition, and intestinal permeability: A systematic review and meta-analysis. Gut Microbes.

[6] Lavelle A., Hoffmann T.W., Pham H.P., Langella P., Guedon E., Sokol H. (2019). Baseline microbiota composition modulates antibiotic-mediated effects on the gut microbiota and host. Microbiome.

[7] Claesson M.J., Jeffery I.B., Conde S., Power S.E., O’Connor E.M., Cusack S., Harris H.M., Coakley M., Lakshminarayanan B., O’Sullivan O. (2012). Gut microbiota composition correlates with diet and health in the elderly. Nature.

[8] Moya A., Ferrer M. (2016). Functional Redundancy-Induced Stability of Gut Microbiota Subjected to Disturbance. Trends Microbiol..

[9] Vieira-Silva S., Falony G., Darzi Y., Lima-Mendez G., Garcia Yunta R., Okuda S., Vandeputte D., Valles-Colomer M., Hildebrand F., Chaffron S. (2016). Species-function relationships shape ecological properties of the human gut microbiome. Nat. Microbiol..

[10] Backhed F., Ding H., Wang T., Hooper L.V., Koh G.Y., Nagy A., Semenkovich C.F., Gordon J.I. (2004). The gut microbiota as an environmental factor that regulates fat storage. Proc. Natl. Acad. Sci. USA.

[11] Schwarzer M., Makki K., Storelli G., Machuca-Gayet I., Srutkova D., Hermanova P., Martino M.E., Balmand S., Hudcovic T., Heddi A. (2016). Lactobacillus plantarum strain maintains growth of infant mice during chronic undernutrition. Science.

[12] Schroeder B.O., Backhed F. (2016). Signals from the gut microbiota to distant organs in physiology and disease. Nat. Med..

[13] Makki K., Deehan E.C., Walter J., Backhed F. (2018). The Impact of Dietary Fiber on Gut Microbiota in Host Health and Disease. Cell Host Microbe.

[14] Round J.L., Mazmanian S.K. (2009). The gut microbiota shapes intestinal immune responses during health and disease. Nat. Rev. Immunol..

[15] Rescigno M. (2014). Intestinal microbiota and its effects on the immune system. Cell Microbiol..

[16] Kamada N., Seo S.U., Chen G.Y., Nunez G. (2013). Role of the gut microbiota in immunity and inflammatory disease. Nat. Rev. Immunol..

[17] Mattner J., Schmidt F., Siegmund B. (2016). Faecal microbiota transplantation-A clinical view. Int. J. Med. Microbiol..

[18] Weingarden A.R., Vaughn B.P. (2017). Intestinal microbiota, fecal microbiota transplantation, and inflammatory bowel disease. Gut Microbes.

[19] Ooijevaar R.E., Terveer E.M., Verspaget H.W., Kuijper E.J., Keller J.J. (2019). Clinical Application and Potential of Fecal Microbiota Transplantation. Annu. Rev. Med..

[20] Sano T., Huang W., Hall J.A., Yang Y., Chen A., Gavzy S.J., Lee J.Y., Ziel J.W., Miraldi E.R., Domingos A.I. (2015). An IL-23R/IL-22 Circuit Regulates Epithelial Serum Amyloid A to Promote Local Effector Th17 Responses. Cell.

[21] Lavelle A., Sokol H. (2020). Gut microbiota-derived metabolites as key actors in inflammatory bowel disease. Nat. Rev. Gastroenterol. Hepatol..

[22] Cani P.D. (2019). Microbiota and metabolites in metabolic diseases. Nat. Rev. Endocrinol..

[23] Parikh K., Antanaviciute A., Fawkner-Corbett D., Jagielowicz M., Aulicino A., Lagerholm C., Davis S., Kinchen J., Chen H.H., Alham N.K. (2019). Colonic epithelial cell diversity in health and inflammatory bowel disease. Nature.

[24] Akdis C.A. (2021). Does the epithelial barrier hypothesis explain the increase in allergy, autoimmunity and other chronic conditions?. Nat. Rev. Immunol..

[25] Zhang Z., Lin T., Meng Y., Hu M., Shu L., Jiang H., Gao R., Ma J., Wang C., Zhou X. (2021). FOS/GOS attenuates high-fat diet induced bone loss via reversing microbiota dysbiosis, high intestinal permeability and systemic inflammation in mice. Metabolism.

[26] Tajik N., Frech M., Schulz O., Schalter F., Lucas S., Azizov V., Durholz K., Steffen F., Omata Y., Rings A. (2020). Targeting zonulin and intestinal epithelial barrier function to prevent onset of arthritis. Nat. Commun..

[27] Monsted M.O., Falck N.D., Pedersen K., Buschard K., Holm L.J., Haupt-Jorgensen M. (2021). Intestinal permeability in type 1 diabetes: An updated comprehensive overview. J. Autoimmun..

[28] Camilleri M. (2019). Leaky gut: Mechanisms, measurement and clinical implications in humans. Gut.

[29] Thevaranjan N., Puchta A., Schulz C., Naidoo A., Szamosi J.C., Verschoor C.P., Loukov D., Schenck L.P., Jury J., Foley K.P. (2018). Age-Associated Microbial Dysbiosis Promotes Intestinal Permeability, Systemic Inflammation, and Macrophage Dysfunction. Cell Host Microbe.

[30] Yu E., Sharma S. (2022). Physiology, Calcium. StatPearls.

[31] Groenendijk I., van Delft M., Versloot P., van Loon L.J.C., de Groot L. (2022). Impact of magnesium on bone health in older adults: A systematic review and meta-analysis. Bone.

[32] Sato H., Takai C., Kazama J.J., Wakamatsu A., Hasegawa E., Kobayashi D., Kondo N., Nakatsue T., Abe A., Ito S. (2020). Serum hepcidin level, iron metabolism and osteoporosis in patients with rheumatoid arthritis. Sci. Rep..

[33] Ma J., Wang A., Zhang H., Liu B., Geng Y., Xu Y., Zuo G., Jia P. (2022). Iron overload induced osteocytes apoptosis and led to bone loss in Hepcidin(-/-) mice through increasing sclerostin and RANKL/OPG. Bone.

[34] Gordon M., Sinopoulou V., Iheozor-Ejiofor Z., Iqbal T., Allen P., Hoque S., Engineer J., Akobeng A.K. (2021). Interventions for treating iron deficiency anaemia in inflammatory bowel disease. Cochrane Database Syst. Rev..

[35] Shah Y., Patel D., Khan N. (2021). Iron deficiency anemia in IBD: An overlooked comorbidity. Expert Rev. Gastroenterol. Hepatol..

[36] Skrypnik K., Suliburska J. (2018). Association between the gut microbiota and mineral metabolism. J. Sci. Food Agric..

[37] Goltzman D. (2018). Functions of vitamin D in bone. Histochem. Cell Biol..

[38] Stallhofer J., Veith L., Diegelmann J., Probst P., Brand S., Schnitzler F., Olszak T., Torok H., Mayerle J., Stallmach A. (2022). Iron Deficiency in Inflammatory Bowel Disease Is Associated With Low Levels of Vitamin D Modulating Serum Hepcidin and Intestinal Ceruloplasmin Expression. Clin. Transl. Gastroenterol..

[39] Singh P., Rawat A., Alwakeel M., Sharif E., Al Khodor S. (2020). The potential role of vitamin D supplementation as a gut microbiota modifier in healthy individuals. Sci. Rep..

[40] Minisola S., Arnold A., Belaya Z., Brandi M.L., Clarke B.L., Hannan F.M., Hofbauer L.C., Insogna K.L., Lacroix A., Liberman U. (2022). Epidemiology, Pathophysiology, and Genetics of Primary Hyperparathyroidism. J. Bone Miner. Res..

[41] Silva B.C. (2022). Skeletal and nonskeletal consequences of hypoparathyroidism. Arch. Endocrinol. Metab..

[42] Xie J., Guo J., Kanwal Z., Wu M., Lv X., Ibrahim N.A., Li P., Buabeid M.A., Arafa E.A., Sun Q. (2020). Calcitonin and Bone Physiology: In Vitro, In Vivo, and Clinical Investigations. Int. J. Endocrinol..

[43] Arpaia N., Campbell C., Fan X., Dikiy S., van der Veeken J., deRoos P., Liu H., Cross J.R., Pfeffer K., Coffer P.J. (2013). Metabolites produced by commensal bacteria promote peripheral regulatory T-cell generation. Nature.

[44] Furusawa Y., Obata Y., Fukuda S., Endo T.A., Nakato G., Takahashi D., Nakanishi Y., Uetake C., Kato K., Kato T. (2013). Commensal microbe-derived butyrate induces the differentiation of colonic regulatory T cells. Nature.

[45] Smith P.M., Howitt M.R., Panikov N., Michaud M., Gallini C.A., Bohlooly Y.M., Glickman J.N., Garrett W.S. (2013). The microbial metabolites, short-chain fatty acids, regulate colonic Treg cell homeostasis. Science.

[46] Tyagi A.M., Yu M., Darby T.M., Vaccaro C., Li J.Y., Owens J.A., Hsu E., Adams J., Weitzmann M.N., Jones R.M. (2018). The Microbial Metabolite Butyrate Stimulates Bone Formation via T Regulatory Cell-Mediated Regulation of WNT10B Expression. Immunity.

[47] Zaiss M.M., Jones R.M., Schett G., Pacifici R. (2019). The gut-bone axis: How bacterial metabolites bridge the distance. J. Clin. Investig..

[48] Yu M., D’Amelio P., Tyagi A.M., Vaccaro C., Li J.Y., Hsu E., Buondonno I., Sassi F., Adams J., Weitzmann M.N. (2018). Regulatory T cells are expanded by Teriparatide treatment in humans and mediate intermittent PTH-induced bone anabolism in mice. EMBO Rep..

[49] Pacifici R. (2021). Role of Gut Microbiota in the Skeletal Response to PTH. J. Clin. Endocrinol. Metab..

[50] Baloun J., Pekacova A., Wenchich L., Hruskova H., Senolt L., Svec X., Pavelka K., Stepan J.J. (2022). Menopausal Transition: Prospective Study of Estrogen Status, Circulating MicroRNAs, and Biomarkers of Bone Metabolism. Front. Endocrinol..

[51] Cheng C.H., Chen L.R., Chen K.H. (2022). Osteoporosis Due to Hormone Imbalance: An Overview of the Effects of Estrogen Deficiency and Glucocorticoid Overuse on Bone Turnover. Int. J. Mol. Sci..

[52] Xu Z., Yu Z., Chen M., Zhang M., Chen R., Yu H., Lin Y., Wang D., Li S., Huang L. (2022). Mechanisms of estrogen deficiency-induced osteoporosis based on transcriptome and DNA methylation. Front. Cell Dev. Biol..

[53] Cenci S., Weitzmann M.N., Roggia C., Namba N., Novack D., Woodring J., Pacifici R. (2000). Estrogen deficiency induces bone loss by enhancing T-cell production of TNF-alpha. J. Clin. Investig..

[54] Baker J.M., Al-Nakkash L., Herbst-Kralovetz M.M. (2017). Estrogen-gut microbiome axis: Physiological and clinical implications. Maturitas.

[55] Lu L., Chen X., Liu Y., Yu X. (2021). Gut microbiota and bone metabolism. FASEB J..

[56] Novince C.M., Whittow C.R., Aartun J.D., Hathaway J.D., Poulides N., Chavez M.B., Steinkamp H.M., Kirkwood K.A., Huang E., Westwater C. (2017). Commensal Gut Microbiota Immunomodulatory Actions in Bone Marrow and Liver have Catabolic Effects on Skeletal Homeostasis in Health. Sci. Rep..

[57] Uchida Y., Irie K., Fukuhara D., Kataoka K., Hattori T., Ono M., Ekuni D., Kubota S., Morita M. (2018). Commensal Microbiota Enhance Both Osteoclast and Osteoblast Activities. Molecules.

[58] Kondo T., Chiba T., Tousen Y. (2022). Short-chain fatty acids, acetate and propionate, directly upregulate osteoblastic differentiation. Int. J. Food Sci. Nutr..

[59] Lucas S., Omata Y., Hofmann J., Bottcher M., Iljazovic A., Sarter K., Albrecht O., Schulz O., Krishnacoumar B., Kronke G. (2018). Short-chain fatty acids regulate systemic bone mass and protect from pathological bone loss. Nat. Commun..

[60] Yao Y., Cai X., Zheng Y., Zhang M., Fei W., Sun D., Zhao M., Ye Y., Zheng C. (2022). Short-chain fatty acids regulate B cells differentiation via the FFA2 receptor to alleviate rheumatoid arthritis. Br. J. Pharmacol..

[61] Al Saedi A., Sharma S., Summers M.A., Nurgali K., Duque G. (2020). The multiple faces of tryptophan in bone biology. Exp. Gerontol..

[62] Gruner N., Mattner J. (2021). Bile Acids and Microbiota: Multifaceted and Versatile Regulators of the Liver-Gut Axis. Int. J. Mol. Sci..

[63] Li L., Rao S., Cheng Y., Zhuo X., Deng C., Xu N., Zhang H., Yang L. (2019). Microbial osteoporosis: The interplay between the gut microbiota and bones via host metabolism and immunity. Microbiologyopen.

[64] Sandoval D.A., D’Alessio D.A. (2015). Physiology of proglucagon peptides: Role of glucagon and GLP-1 in health and disease. Physiol. Rev..

[65] Adachi R., Honma Y., Masuno H., Kawana K., Shimomura I., Yamada S., Makishima M. (2005). Selective activation of vitamin D receptor by lithocholic acid acetate, a bile acid derivative. J. Lipid Res..

[66] de Souza H.S., Fiocchi C. (2016). Immunopathogenesis of IBD: Current state of the art. Nat. Rev. Gastroenterol. Hepatol..

[67] Garrett W.S., Gordon J.I., Glimcher L.H. (2010). Homeostasis and inflammation in the intestine. Cell.

[68] Conrad K., Roggenbuck D., Laass M.W. (2014). Diagnosis and classification of ulcerative colitis. Autoimmun. Rev..

[69] Laass M.W., Roggenbuck D., Conrad K. (2014). Diagnosis and classification of Crohn’s disease. Autoimmun. Rev..

[70] Maloy K.J., Powrie F. (2011). Intestinal homeostasis and its breakdown in inflammatory bowel disease. Nature.

[71] Neurath M.F. (2014). Cytokines in inflammatory bowel disease. Nat. Rev. Immunol..

[72] Abraham C., Cho J.H. (2009). Inflammatory bowel disease. N. Engl. J. Med..

[73] Atreya R., Neurath M.F. (2015). IBD pathogenesis in 2014: Molecular pathways controlling barrier function in IBD. Nat. Rev. Gastroenterol. Hepatol..

[74] Langer V., Vivi E., Regensburger D., Winkler T.H., Waldner M.J., Rath T., Schmid B., Skottke L., Lee S., Jeon N.L. (2019). IFN-gamma drives inflammatory bowel disease pathogenesis through VE-cadherin-directed vascular barrier disruption. J. Clin. Investig..

[75] Clark M., Colombel J.F., Feagan B.C., Fedorak R.N., Hanauer S.B., Kamm M.A., Mayer L., Regueiro C., Rutgeerts P., Sandborn W.J. (2007). American gastroenterological association consensus development conference on the use of biologics in the treatment of inflammatory bowel disease, June 21-23, 2006. Gastroenterology.

[76] Atreya R., Zimmer M., Bartsch B., Waldner M.J., Atreya I., Neumann H., Hildner K., Hoffman A., Kiesslich R., Rink A.D. (2011). Antibodies against tumor necrosis factor (TNF) induce T-cell apoptosis in patients with inflammatory bowel diseases via TNF receptor 2 and intestinal CD14(+) macrophages. Gastroenterology.

[77] Atreya R., Neumann H., Neufert C., Waldner M.J., Billmeier U., Zopf Y., Willma M., App C., Munster T., Kessler H. (2014). In vivo imaging using fluorescent antibodies to tumor necrosis factor predicts therapeutic response in Crohn’s disease. Nat. Med..

[78] Packey C.D., Sartor R.B. (2009). Commensal bacteria, traditional and opportunistic pathogens, dysbiosis and bacterial killing in inflammatory bowel diseases. Curr. Opin. Infect. Dis..

[79] Reiff C., Kelly D. (2010). Inflammatory bowel disease, gut bacteria and probiotic therapy. Int. J. Med. Microbiol. IJMM.

[80] Sartor R.B. (2008). Microbial influences in inflammatory bowel diseases. Gastroenterology.

[81] Clavel T., Gomes-Neto J.C., Lagkouvardos I., Ramer-Tait A.E. (2017). Deciphering interactions between the gut microbiota and the immune system via microbial cultivation and minimal microbiomes. Immunol. Rev..

[82] Yoo J.Y., Groer M., Dutra S.V.O., Sarkar A., McSkimming D.I. (2020). Gut Microbiota and Immune System Interactions. Microorganisms.

[83] Yue B., Luo X., Yu Z., Mani S., Wang Z., Dou W. (2019). Inflammatory Bowel Disease: A Potential Result from the Collusion between Gut Microbiota and Mucosal Immune System. Microorganisms.

[84] Brown H., Esterhazy D. (2021). Intestinal immune compartmentalization: Implications of tissue specific determinants in health and disease. Mucosal Immunol..

[85] Hill D.A., Artis D. (2010). Intestinal bacteria and the regulation of immune cell homeostasis. Annu. Rev. Immunol..

[86] Fang H., Fu L., Wang J. (2018). Protocol for Fecal Microbiota Transplantation in Inflammatory Bowel Disease: A Systematic Review and Meta-Analysis. Biomed Res. Int..

[87] Imdad A., Nicholson M.R., Tanner-Smith E.E., Zackular J.P., Gomez-Duarte O.G., Beaulieu D.B., Acra S. (2018). Fecal transplantation for treatment of inflammatory bowel disease. Cochrane Database Syst. Rev..

[88] Stojek M., Jablonska A., Adrych K. (2021). The Role of Fecal Microbiota Transplantation in the Treatment of Inflammatory Bowel Disease. J. Clin. Med..

[89] Tan P., Li X., Shen J., Feng Q. (2020). Fecal Microbiota Transplantation for the Treatment of Inflammatory Bowel Disease: An Update. Front. Pharmacol..

[90] Sands B.E. (2004). From symptom to diagnosis: Clinical distinctions among various forms of intestinal inflammation. Gastroenterology.

[91] Grimstad T., Norheim K.B., Isaksen K., Leitao K., Hetta A.K., Carlsen A., Karlsen L.N., Skoie I.M., Goransson L., Harboe E. (2015). Fatigue in Newly Diagnosed Inflammatory Bowel Disease. J. Crohns Colitis.

[92] Hedin C.R.H., Vavricka S.R., Stagg A.J., Schoepfer A., Raine T., Puig L., Pleyer U., Navarini A., van der Meulen-de Jong A.E., Maul J. (2019). The Pathogenesis of Extraintestinal Manifestations: Implications for IBD Research, Diagnosis, and Therapy. J. Crohns Colitis.

[93] Rogler G., Singh A., Kavanaugh A., Rubin D.T. (2021). Extraintestinal Manifestations of Inflammatory Bowel Disease: Current Concepts, Treatment, and Implications for Disease Management. Gastroenterology.

[94] Malik T.F., Aurelio D.M. (2022). Extraintestinal Manifestations of Inflammatory Bowel Disease. StatPearls.

[95] Barkhodari A., Lee K.E., Shen M., Shen B., Yao Q. (2022). Inflammatory Bowel Disease: Focus on Enteropathic Arthritis and Therapy. Rheumatol. Immunol. Res..

[96] Ashrafi M., Kuhn K.A., Weisman M.H. (2021). The arthritis connection to inflammatory bowel disease (IBD): Why has it taken so long to understand it?. RMD Open.

[97] Karreman M.C., Luime J.J., Hazes J.M.W., Weel A. (2017). The Prevalence and Incidence of Axial and Peripheral Spondyloarthritis in Inflammatory Bowel Disease: A Systematic Review and Meta-analysis. J. Crohns Colitis.

[98] Healy P.J., Helliwell P.S. (2005). Classification of the spondyloarthropathies. Curr. Opin. Rheumatol..

[99] Sen R., Goyal A., Hurley J.A. (2022). Seronegative Spondyloarthropathy. StatPearls.

[100] Dougados M., Baeten D. (2011). Spondyloarthritis. Lancet.

[101] Ensrud K.E., Crandall C.J. (2017). Osteoporosis. Ann. Intern. Med..

[102] Feng X., McDonald J.M. (2011). Disorders of bone remodeling. Annu. Rev. Pathol..

[103] Foger-Samwald U., Dovjak P., Azizi-Semrad U., Kerschan-Schindl K., Pietschmann P. (2020). Osteoporosis: Pathophysiology and therapeutic options. EXCLI J..

[104] Varacallo M., Seaman T.J., Jandu J.S., Pizzutillo P. (2022). Osteopenia. StatPearls.

[105] Porter J.L., Varacallo M. (2022). Osteoporosis. StatPearls.

[106] Zaidi M. (2007). Skeletal remodeling in health and disease. Nat. Med..

[107] Xu X., Jia X., Mo L., Liu C., Zheng L., Yuan Q., Zhou X. (2017). Intestinal microbiota: A potential target for the treatment of postmenopausal osteoporosis. Bone Res..

[108] Wen K., Tao L., Tao Z., Meng Y., Zhou S., Chen J., Yang K., Da W., Zhu Y. (2020). Fecal and Serum Metabolomic Signatures and Microbial Community Profiling of Postmenopausal Osteoporosis Mice Model. Front. Cell Infect. Microbiol..

[109] Rettedal E.A., Ilesanmi-Oyelere B.L., Roy N.C., Coad J., Kruger M.C. (2021). The Gut Microbiome Is Altered in Postmenopausal Women With Osteoporosis and Osteopenia. JBMR Plus.

[110] Yang X., Chang T., Yuan Q., Wei W., Wang P., Song X., Yuan H. (2022). Changes in the composition of gut and vaginal microbiota in patients with postmenopausal osteoporosis. Front. Immunol..

[111] Kurabayashi T., Fujimaki T., Yasuda M., Yamamoto Y., Tanaka K. (1993). Time-course of vertebral and femoral bone loss in rats administered gonadotrophin-releasing hormone agonist. J. Endocrinol..

[112] Wang Y., Yano T., Kikuchi A., Yano N., Matsumi H., Ando K., Kasai Y., Watanabe M., Okagaki R., Osuga Y. (2000). Comparison of the effects of add-back therapy with various natural oestrogens on bone metabolism in rats administered a long-acting gonadotrophin-releasing hormone agonist. J. Endocrinol..

[113] Li J.Y., Chassaing B., Tyagi A.M., Vaccaro C., Luo T., Adams J., Darby T.M., Weitzmann M.N., Mulle J.G., Gewirtz A.T. (2016). Sex steroid deficiency-associated bone loss is microbiota dependent and prevented by probiotics. J. Clin. Investig..

[114] Zhang Y.W., Cao M.M., Li Y.J., Lu P.P., Dai G.C., Zhang M., Wang H., Rui Y.F. (2022). Fecal microbiota transplantation ameliorates bone loss in mice with ovariectomy-induced osteoporosis via modulating gut microbiota and metabolic function. J. Orthop. Translat..

[115] Krela-Kazmierczak I., Szymczak A., Lykowska-Szuber L., Eder P., Linke K. (2016). Osteoporosis in Gastrointestinal Diseases. Adv. Clin. Exp. Med..

[116] Abe K., Takahashi A., Imaizumi H., Hayashi M., Okai K., Kanno Y., Sato N., Kenjo A., Marubashi S., Ohira H. (2018). Utility of magnetic resonance elastography for predicting ascites in patients with chronic liver disease. J. Gastroenterol. Hepatol..

[117] Hayashi M., Abe K., Fujita M., Okai K., Takahashi A., Ohira H. (2018). Association between sarcopenia and osteoporosis in chronic liver disease. Hepatol. Res..

[118] Szafors P., Che H., Barnetche T., Morel J., Gaujoux-Viala C., Combe B., Lukas C. (2018). Risk of fracture and low bone mineral density in adults with inflammatory bowel diseases. A systematic literature review with meta-analysis. Osteoporos. Int..

[119] Tulewicz-Marti E.M., Lewandowski K., Rydzewska G. (2022). Bone Metabolism Alteration in Patients with Inflammatory Bowel Disease. J. Clin. Med..

[120] Katz S. (2006). Osteoporosis in patients with inflammatory bowel disease: Risk factors, prevention, and treatment. Rev. Gastroenterol. Disord..

[121] Lima C.A., Lyra A.C., Rocha R., Santana G.O. (2015). Risk factors for osteoporosis in inflammatory bowel disease patients. World J. Gastrointest. Pathophysiol..

[122] Lo B., Holm J.P., Vester-Andersen M.K., Bendtsen F., Vind I., Burisch J. (2020). Incidence, Risk Factors and Evaluation of Osteoporosis in Patients With Inflammatory Bowel Disease: A Danish Population-Based Inception Cohort With 10 Years of Follow-Up. J. Crohns Colitis.

[123] Ali T., Lam D., Bronze M.S., Humphrey M.B. (2009). Osteoporosis in inflammatory bowel disease. Am. J. Med..

[124] Fernandes B.M., Rosa-Goncalves D., Magro F., Costa L., Bernardes M. (2022). Musculoskeletal manifestations in a Portuguese cohort of 235 inflammatory bowel disease patients. ARP Rheumatol..

[125] Parizadeh S.M., Jafarzadeh-Esfehani R., Hassanian S.M., Mottaghi-Moghaddam A., Ghazaghi A., Ghandehari M., Alizade-Noghani M., Khazaei M., Ghayour-Mobarhan M., Ferns G.A. (2019). Vitamin D in inflammatory bowel disease: From biology to clinical implications. Complement. Ther. Med..

[126] Garber A., Regueiro M. (2019). Extraintestinal Manifestations of Inflammatory Bowel Disease: Epidemiology, Etiopathogenesis, and Management. Curr. Gastroenterol. Rep..

[127] Sylvester F.A. (2017). Inflammatory Bowel Disease: Effects on Bone and Mechanisms. Adv. Exp. Med. Biol..

[128] Ezzat Y., Hamdy K. (2010). The frequency of low bone mineral density and its associated risk factors in patients with inflammatory bowel diseases. Int. J. Rheum. Dis..

[129] Jahnsen J., Falch J.A., Aadland E., Mowinckel P. (1997). Bone mineral density is reduced in patients with Crohn’s disease but not in patients with ulcerative colitis: A population based study. Gut.

[130] Lima C.A., Lyra A.C., Mendes C.M.C., Lopes M.B., Coqueiro F.G., Rocha R., Santana G.O. (2017). Bone mineral density and inflammatory bowel disease severity. Braz. J. Med. Biol. Res..

[131] Maratova K., Hradsky O., Matyskova J., Copova I., Soucek O., Sumnik Z., Bronsky J. (2017). Musculoskeletal system in children and adolescents with inflammatory bowel disease: Normal muscle force, decreased trabecular bone mineral density and low prevalence of vertebral fractures. Eur. J. Pediatr..

[132] Even Dar R., Mazor Y., Karban A., Ish-Shalom S., Segal E. (2019). Risk Factors for Low Bone Density in Inflammatory Bowel Disease: Use of Glucocorticoids, Low Body Mass Index, and Smoking. Dig. Dis..

[133] Bernstein C.N., Benchimol E.I., Bitton A., Murthy S.K., Nguyen G.C., Lee K., Cooke-Lauder J., Kaplan G.G. (2019). The Impact of Inflammatory Bowel Disease in Canada 2018: Extra-intestinal Diseases in IBD. J. Can. Assoc. Gastroenterol..

[134] Janssen K.M.J., Hop H., Vissink A., Dijkstra G., de Smit M.J., Brouwer E., Westra J. (2020). Levels of Anti-Citrullinated Protein Antibodies and Rheumatoid Factor, Including IgA Isotypes, and Articular Manifestations in Ulcerative Colitis and Crohn’s Disease. Int. J. Environ. Res. Public Health.

[135] Ottaviani S., Treton X., Forien M., Coralli R., Dauchez A., Stefanescu C., Pelletier A.L., Becheur H., Ebstein E., Bouhnik Y. (2022). Screening for spondyloarthritis in patients with inflammatory bowel diseases. Rheumatol. Int..

[136] Rahmani P., Rasti G., Gorgi M., Motamed F., Sharifi P. (2022). Extraintestinal manifestation of inflammatory bowel disease and associated factors in pediatric patients. Ann. Med. Surg..

[137] Epsley S., Tadros S., Farid A., Kargilis D., Mehta S., Rajapakse C.S. (2020). The Effect of Inflammation on Bone. Front. Physiol..

[138] Ding P., Tan Q., Wei Z., Chen Q., Wang C., Qi L., Wen L., Zhang C., Yao C. (2022). Toll-like receptor 9 deficiency induces osteoclastic bone loss via gut microbiota-associated systemic chronic inflammation. Bone Res..

[139] Al Saedi A., Sharma S., Bani Hassan E., Chen L., Ghasem-Zadeh A., Hassanzadeganroudsari M., Gooi J.H., Stavely R., Eri R., Miao D. (2022). Characterization of Skeletal Phenotype and Associated Mechanisms With Chronic Intestinal Inflammation in the Winnie Mouse Model of Spontaneous Chronic Colitis. Inflamm. Bowel Dis..

[140] Sternes P.R., Brett L., Phipps J., Ciccia F., Kenna T., de Guzman E., Zimmermann K., Morrison M., Holtmann G., Klingberg E. (2022). Distinctive gut microbiomes of ankylosing spondylitis and inflammatory bowel disease patients suggest differing roles in pathogenesis and correlate with disease activity. Arthritis. Res. Ther..

[141] Muniz Pedrogo D.A., Chen J., Hillmann B., Jeraldo P., Al-Ghalith G., Taneja V., Davis J.M., Knights D., Nelson H., Faubion W.A. (2019). An Increased Abundance of Clostridiaceae Characterizes Arthritis in Inflammatory Bowel Disease and Rheumatoid Arthritis: A Cross-sectional Study. Inflamm. Bowel Dis..

[142] Forbes J.D., Chen C.Y., Knox N.C., Marrie R.A., El-Gabalawy H., de Kievit T., Alfa M., Bernstein C.N., Van Domselaar G. (2018). A comparative study of the gut microbiota in immune-mediated inflammatory diseases-does a common dysbiosis exist?. Microbiome.

[143] Ma H., Tao W., Zhu S. (2019). T lymphocytes in the intestinal mucosa: Defense and tolerance. Cell Mol. Immunol..

[144] Schreurs R., Sagebiel A.F., Steinert F.L., Highton A.J., Klarenbeek P.L., Drewniak A., Bakx R., The S.M.L., Ribeiro C.M.S., Perez D. (2021). Intestinal CD8(+) T cell responses are abundantly induced early in human development but show impaired cytotoxic effector capacities. Mucosal Immunol..

[145] Zaiss M.M., Joyce Wu H.J., Mauro D., Schett G., Ciccia F. (2021). The gut-joint axis in rheumatoid arthritis. Nat. Rev. Rheumatol..

[146] Fan Z., Ross R.P., Stanton C., Hou B., Zhao J., Zhang H., Yang B., Chen W. (2021). Lactobacillus casei CCFM1074 Alleviates Collagen-Induced Arthritis in Rats via Balancing Treg/Th17 and Modulating the Metabolites and Gut Microbiota. Front. Immunol..

[147] Rogier R., Ederveen T.H.A., Boekhorst J., Wopereis H., Scher J.U., Manasson J., Frambach S., Knol J., Garssen J., van der Kraan P.M. (2017). Aberrant intestinal microbiota due to IL-1 receptor antagonist deficiency promotes IL-17- and TLR4-dependent arthritis. Microbiome.

[148] Rogier R., Evans-Marin H., Manasson J., van der Kraan P.M., Walgreen B., Helsen M.M., van den Bersselaar L.A., van de Loo F.A., van Lent P.L., Abramson S.B. (2017). Alteration of the intestinal microbiome characterizes preclinical inflammatory arthritis in mice and its modulation attenuates established arthritis. Sci. Rep..

[149] Yu M., Malik Tyagi A., Li J.Y., Adams J., Denning T.L., Weitzmann M.N., Jones R.M., Pacifici R. (2020). PTH induces bone loss via microbial-dependent expansion of intestinal TNF(+) T cells and Th17 cells. Nat. Commun..

[150] Tanaka S., Nakamura K., Takahasi N., Suda T. (2005). Role of RANKL in physiological and pathological bone resorption and therapeutics targeting the RANKL-RANK signaling system. Immunol. Rev..

[151] Chen D.Y., Chen Y.M., Chen H.H., Hsieh C.W., Lin C.C., Lan J.L. (2011). Increasing levels of circulating Th17 cells and interleukin-17 in rheumatoid arthritis patients with an inadequate response to anti-TNF-alpha therapy. Arthritis Res. Ther..

[152] Lam J., Takeshita S., Barker J.E., Kanagawa O., Ross F.P., Teitelbaum S.L. (2000). TNF-alpha induces osteoclastogenesis by direct stimulation of macrophages exposed to permissive levels of RANK ligand. J. Clin. Investig..

[153] Wu S., Liao A.P., Xia Y., Li Y.C., Li J.D., Sartor R.B., Sun J. (2010). Vitamin D receptor negatively regulates bacterial-stimulated NF-kappaB activity in intestine. Am. J. Pathol..

[154] Torres J., Petralia F., Sato T., Wang P., Telesco S.E., Choung R.S., Strauss R., Li X.J., Laird R.M., Gutierrez R.L. (2020). Serum Biomarkers Identify Patients Who Will Develop Inflammatory Bowel Diseases Up to 5 Years Before Diagnosis. Gastroenterology.

[155] Turpin W., Lee S.H., Raygoza Garay J.A., Madsen K.L., Meddings J.B., Bedrani L., Power N., Espin-Garcia O., Xu W., Smith M.I. (2020). Increased Intestinal Permeability Is Associated With Later Development of Crohn’s Disease. Gastroenterology.

[156] Mehandru S., Colombel J.F. (2021). The intestinal barrier, an arbitrator turned provocateur in IBD. Nat. Rev. Gastroenterol. Hepatol..

[157] Luchetti M.M., Ciccia F., Avellini C., Benfaremo D., Rizzo A., Spadoni T., Svegliati S., Marzioni D., Santinelli A., Costantini A. (2021). Gut epithelial impairment, microbial translocation and immune system activation in inflammatory bowel disease-associated spondyloarthritis. Rheumatology.

[158] Berlinberg A., Kuhn K.A. (2020). Barrier lymphocytes in spondyloarthritis. Curr. Opin. Rheumatol..

[159] Lefferts A.R., Norman E., Claypool D.J., Kantheti U., Kuhn K.A. (2022). Cytokine competent gut-joint migratory T Cells contribute to inflammation in the joint. Front. Immunol..

[160] Salmi M., Andrew D.P., Butcher E.C., Jalkanen S. (1995). Dual binding capacity of mucosal immunoblasts to mucosal and synovial endothelium in humans: Dissection of the molecular mechanisms. J. Exp. Med..

[161] Salmi M., Rajala P., Jalkanen S. (1997). Homing of mucosal leukocytes to joints. Distinct endothelial ligands in synovium mediate leukocyte-subtype specific adhesion. J. Clin. Investig..

[162] Salmi M., Jalkanen S. (2001). Human leukocyte subpopulations from inflamed gut bind to joint vasculature using distinct sets of adhesion molecules. J. Immunol..

[163] Talmor-Barkan Y., Bar N., Shaul A.A., Shahaf N., Godneva A., Bussi Y., Lotan-Pompan M., Weinberger A., Shechter A., Chezar-Azerrad C. (2022). Metabolomic and microbiome profiling reveals personalized risk factors for coronary artery disease. Nat. Med..

[164] van den Munckhof I.C.L., Kurilshikov A., Ter Horst R., Riksen N.P., Joosten L.A.B., Zhernakova A., Fu J., Keating S.T., Netea M.G., de Graaf J. (2018). Role of gut microbiota in chronic low-grade inflammation as potential driver for atherosclerotic cardiovascular disease: A systematic review of human studies. Obes. Rev..

[165] Yasar Bilge N.S., Perez Brocal V., Kasifoglu T., Bilge U., Kasifoglu N., Moya A., Dinleyici E.C. (2020). Intestinal microbiota composition of patients with Behcet’s disease: Differences between eye, mucocutaneous and vascular involvement. The Rheuma-BIOTA study. Clin. Exp. Rheumatol..

[166] Andriessen E.M., Wilson A.M., Mawambo G., Dejda A., Miloudi K., Sennlaub F., Sapieha P. (2016). Gut microbiota influences pathological angiogenesis in obesity-driven choroidal neovascularization. EMBO Mol. Med..

[167] Spadoni I., Zagato E., Bertocchi A., Paolinelli R., Hot E., Di Sabatino A., Caprioli F., Bottiglieri L., Oldani A., Viale G. (2015). A gut-vascular barrier controls the systemic dissemination of bacteria. Science.

[168] Karbach S.H., Schonfelder T., Brandao I., Wilms E., Hormann N., Jackel S., Schuler R., Finger S., Knorr M., Lagrange J. (2016). Gut Microbiota Promote Angiotensin II-Induced Arterial Hypertension and Vascular Dysfunction. J. Am. Heart Assoc..

[169] Scaldaferri F., Vetrano S., Sans M., Arena V., Straface G., Stigliano E., Repici A., Sturm A., Malesci A., Panes J. (2009). VEGF-A links angiogenesis and inflammation in inflammatory bowel disease pathogenesis. Gastroenterology.

[170] Alkim C., Alkim H., Koksal A.R., Boga S., Sen I. (2015). Angiogenesis in Inflammatory Bowel Disease. Int. J. Inflam..

[171] Eder P., Korybalska K., Linke K., Witowski J. (2015). Angiogenesis-related proteins--their role in the pathogenesis and treatment of inflammatory bowel disease. Curr. Protein Pept. Sci..

[172] Joe B., McCarthy C.G., Edwards J.M., Cheng X., Chakraborty S., Yang T., Golonka R.M., Mell B., Yeo J.Y., Bearss N.R. (2020). Microbiota Introduced to Germ-Free Rats Restores Vascular Contractility and Blood Pressure. Hypertension.

[173] Vicentini F.A., Keenan C.M., Wallace L.E., Woods C., Cavin J.B., Flockton A.R., Macklin W.B., Belkind-Gerson J., Hirota S.A., Sharkey K.A. (2021). Intestinal microbiota shapes gut physiology and regulates enteric neurons and glia. Microbiome.

[174] Yoo B.B., Mazmanian S.K. (2017). The Enteric Network: Interactions between the Immune and Nervous Systems of the Gut. Immunity.

[175] Spencer N.J., Hu H. (2020). Enteric nervous system: Sensory transduction, neural circuits and gastrointestinal motility. Nat. Rev. Gastroenterol. Hepatol..

[176] Gulbransen B.D., Sharkey K.A. (2012). Novel functional roles for enteric glia in the gastrointestinal tract. Nat. Rev. Gastroenterol. Hepatol..

[177] Sharkey K.A., Beck P.L., McKay D.M. (2018). Neuroimmunophysiology of the gut: Advances and emerging concepts focusing on the epithelium. Nat. Rev. Gastroenterol. Hepatol..

[178] Furness J.B. (2012). The enteric nervous system and neurogastroenterology. Nat. Rev. Gastroenterol. Hepatol..

[179] Kennedy A., Ng C.T., Biniecka M., Saber T., Taylor C., O’Sullivan J., Veale D.J., Fearon U. (2010). Angiogenesis and blood vessel stability in inflammatory arthritis. Arthritis Rheum..

[180] Maeda Y., Kurakawa T., Umemoto E., Motooka D., Ito Y., Gotoh K., Hirota K., Matsushita M., Furuta Y., Narazaki M. (2016). Dysbiosis Contributes to Arthritis Development via Activation of Autoreactive T Cells in the Intestine. Arthritis Rheumatol..

[181] Panfili E., Gerli R., Grohmann U., Pallotta M.T. (2020). Amino Acid Metabolism in Rheumatoid Arthritis: Friend or Foe?. Biomolecules.

[182] Scoville E.A., Allaman M.M., Brown C.T., Motley A.K., Horst S.N., Williams C.S., Koyama T., Zhao Z., Adams D.W., Beaulieu D.B. (2018). Alterations in Lipid, Amino Acid, and Energy Metabolism Distinguish Crohn’s Disease from Ulcerative Colitis and Control Subjects by Serum Metabolomic Profiling. Metabolomics.

[183] Coburn L.A., Horst S.N., Allaman M.M., Brown C.T., Williams C.S., Hodges M.E., Druce J.P., Beaulieu D.B., Schwartz D.A., Wilson K.T. (2016). L-Arginine Availability and Metabolism Is Altered in Ulcerative Colitis. Inflamm. Bowel Dis..

[184] Horowitz S., Binion D.G., Nelson V.M., Kanaa Y., Javadi P., Lazarova Z., Andrekopoulos C., Kalyanaraman B., Otterson M.F., Rafiee P. (2007). Increased arginase activity and endothelial dysfunction in human inflammatory bowel disease. Am. J. Physiol. Gastrointest. Liver Physiol..

[185] Li J.Y., Guo Y.C., Zhou H.F., Yue T.T., Wang F.X., Sun F., Wang W.Z. (2022). Arginine metabolism regulates the pathogenesis of inflammatory bowel disease. Nutr. Rev..

[186] Baier J., Gansbauer M., Giessler C., Arnold H., Muske M., Schleicher U., Lukassen S., Ekici A., Rauh M., Daniel C. (2020). Arginase impedes the resolution of colitis by altering the microbiome and metabolome. J. Clin. Investig..

[187] Coburn L.A., Gong X., Singh K., Asim M., Scull B.P., Allaman M.M., Williams C.S., Rosen M.J., Washington M.K., Barry D.P. (2012). L-arginine supplementation improves responses to injury and inflammation in dextran sulfate sodium colitis. PLoS ONE.

[188] Andrade M.E., Santos R.D., Soares A.D., Costa K.A., Fernandes S.O., de Souza C.M., Cassali G.D., de Souza A.L., Faria A.M., Cardoso V.N. (2016). Pretreatment and Treatment with L-Arginine Attenuate Weight Loss and Bacterial Translocation in Dextran Sulfate Sodium Colitis. JPEN J. Parenter. Enter. Nutr..

[189] Singh K., Gobert A.P., Coburn L.A., Barry D.P., Allaman M., Asim M., Luis P.B., Schneider C., Milne G.L., Boone H.H. (2019). Dietary Arginine Regulates Severity of Experimental Colitis and Affects the Colonic Microbiome. Front. Cell Infect. Microbiol..

[190] Stettner N., Rosen C., Bernshtein B., Gur-Cohen S., Frug J., Silberman A., Sarver A., Carmel-Neiderman N.N., Eilam R., Biton I. (2018). Induction of Nitric-Oxide Metabolism in Enterocytes Alleviates Colitis and Inflammation-Associated Colon Cancer. Cell Rep..

[191] Huang H.L., Chen W.C., Hsu H.P., Cho C.Y., Hung Y.H., Wang C.Y., Lai M.D. (2017). Silencing of argininosuccinate lyase inhibits colorectal cancer formation. Oncol. Rep..

[192] Zhu J., Wang T., Lin Y., Xiong M., Chen J., Jian C., Zhang J., Xie H., Zeng F., Huang Q. (2022). The change of plasma metabolic profile and gut microbiome dysbiosis in patients with rheumatoid arthritis. Front. Microbiol..

[193] Brunner J.S., Vulliard L., Hofmann M., Kieler M., Lercher A., Vogel A., Russier M., Bruggenthies J.B., Kerndl M., Saferding V. (2020). Environmental arginine controls multinuclear giant cell metabolism and formation. Nat. Commun..

[194] Hannemann N., Cao S., Eriksson D., Schnelzer A., Jordan J., Eberhardt M., Schleicher U., Rech J., Ramming A., Uebe S. (2019). Transcription factor Fra-1 targets arginase-1 to enhance macrophage-mediated inflammation in arthritis. J. Clin. Investig..

[195] Zeisel S.H., Warrier M. (2017). Trimethylamine N-Oxide, the Microbiome, and Heart and Kidney Disease. Annu. Rev. Nutr..

[196] Zhou T., Heianza Y., Chen Y., Li X., Sun D., DiDonato J.A., Pei X., LeBoff M.S., Bray G.A., Sacks F.M. (2019). Circulating Gut Microbiota Metabolite Trimethylamine N-Oxide (TMAO) and Changes in Bone Density in Response to Weight Loss Diets: The POUNDS Lost Trial. Diabetes Care.

[197] Peng J., Yu X.J., Yu L.L., Tian F.W., Zhao J.X., Zhang H., Chen W., Zhai Q.X. (2021). The influence of gut microbiome on bone health and related dietary strategies against bone dysfunctions. Food Res. Int..

[198] Turner D., Ricciuto A., Lewis A., D’Amico F., Dhaliwal J., Griffiths A.M., Bettenworth D., Sandborn W.J., Sands B.E., Reinisch W. (2021). STRIDE-II: An Update on the Selecting Therapeutic Targets in Inflammatory Bowel Disease (STRIDE) Initiative of the International Organization for the Study of IBD (IOIBD): Determining Therapeutic Goals for Treat-to-Target strategies in IBD. Gastroenterology.

[199] Torres J., Bonovas S., Doherty G., Kucharzik T., Gisbert J.P., Raine T., Adamina M., Armuzzi A., Bachmann O., Bager P. (2020). ECCO Guidelines on Therapeutics in Crohn’s Disease: Medical Treatment. J. Crohns Colitis.

[200] Chen M., Feng Y., Liu W. (2021). Efficacy and safety of probiotics in the induction and maintenance of inflammatory bowel disease remission: A systematic review and meta-analysis. Ann. Palliat. Med..

[201] Kelly C.R., Yen E.F., Grinspan A.M., Kahn S.A., Atreja A., Lewis J.D., Moore T.A., Rubin D.T., Kim A.M., Serra S. (2021). Fecal Microbiota Transplantation Is Highly Effective in Real-World Practice: Initial Results From the FMT National Registry. Gastroenterology.

[202] Zhang L., Ma X., Liu P., Ge W., Hu L., Zuo Z., Xiao H., Liao W. (2021). Treatment and mechanism of fecal microbiota transplantation in mice with experimentally induced ulcerative colitis. Exp. Biol. Med..

[203] Green J.E., Davis J.A., Berk M., Hair C., Loughman A., Castle D., Athan E., Nierenberg A.A., Cryan J.F., Jacka F. (2020). Efficacy and safety of fecal microbiota transplantation for the treatment of diseases other than Clostridium difficile infection: A systematic review and meta-analysis. Gut Microbes.

[204] Agrawal M., Arora S., Li J., Rahmani R., Sun L., Steinlauf A.F., Mechanick J.I., Zaidi M. (2011). Bone, inflammation, and inflammatory bowel disease. Curr. Osteoporos. Rep..

